# Notes and News

**Published:** 1889-06-22

**Authors:** 


### Notes and News.

Collected and Communicated.

The annual meeting of the After-Care Association for Poor and Friendless Female Convalescents on Leaving Asylums for the Insane will take place at 83, Lancaster-gate, on Thursday, July 4, at 3 p.m., when the Earl of Meath will preside.

A series of lectures to medical men are being given on Tuesdays and Friday at the Sanitary Institute. The first was held yesterday (Friday), when Mr. Brudenell Carter discoursed on "Some Considerations on Ocular Hygiene." On Tuesday next, Dr. Edward Seaton will lecture on the Infectious Hospitals of London; and amongst others who will take part in the course are Dr. Louis Parkes, Professor Corfield, and Sir Douglas Galton.

The annual meeting of governors of Dundee Royal Infirmary was held on June 10, Mr. R. G. Kennedy in the chair. The secretary read the medical report, which set forth that during the year, which ended with May 15, 1889, there were admitted into the infirmary 1,839 patients, being 213 less than in 1888, and 397 less than in 1887. In the dispensary the number of teeth extracted was 4,077. Moreover, the district surgeons attended, at their homes when necessary, 5,893 patients, all of whom were supplied with medicines at the expense of the infirmary. The convalescent home at Barnhill continues to form a valuable auxiliary to the infirmary. The total admissions this year have been 934, as against 989, showing a decrease of 55. Mr. Robert Sturrock reported that the revenue for the year amounted to £7,231 13s., and the expenditure to £7,083 15s. 9d., leaving a balance of £147 17s. 3d. in favour of the institution.

The great gala and bazaar in aid of the Stanley Hospital, Liverpool, held last week, was, as was expected, an enormous success. The lady stall-holders and assistants number 150, and their costumes were as diverse as they were picturesque. The Mayor of Liverpool, in opening the bazaar, said that the hospital was founded in 1867, and the building, which was now so prominent an object in Stanley-road, was erected in 1872. Having been in existence only some sixteen years, it was affording relief to the suffering to the number of 41,000 a year. Surely, no further or stronger argument was required to enlist their sympathy and support. The object of their presence being to increase its means of usefulness, he could only say with what pride and satisfaction he looked upon such a large gathering eager for the success of the work. Over £4,000 was wanted to clear off the debt, and they also wanted to put a large amount to the credit of the hospital. He hoped they would put their shoulders to the wheel and exceed all their former efforts.

The committee who have undertaken the erection of a Cottage Hospital at Norwood have issued their first report. The estimated cost of the building designed for six beds is £1,000, and £375 yet remains to be subscribed of this sum. The whole cost of furnishing the hospital has been generously undertaken by friends, and Mr. A. Henderson has given a suitable site. Mr. C. J. Mann has been appointed architect, and has designed a very picturesque little cottage, as much attention, however, being paid to sanitation and ventilation as to beauty.

The Hospital Saturday Committee at Leamington can congratulate themselves on their last year's work, which yielded to the Warneford Hospital a sum of £287, being an increase of £7 on the previous year's collection. The Mayor of Leamington and Dr. Thursfield lately spoke eloquently on the good done by the fund, and the Mayor promised that his wife should this year act as one of the collectors. Women shine in this position, and it is strange that the ladies of Leamington have not before offered their personal aid. The Warneford Hospital is one worthy of the support of rich and poor alike.

Miss Mary Grenfell lately laid the foundation-stone of a new eye hospital for Swansea. The building is situated close to the general hospital premises, with which it will be connected by a covered corridor. The main entrance is from St. Helens-road, and facing this road is a porch, from which is entered the main hall, which is to be used also as a waiting-room for out-patients. From this hall diverge the consulting-room, operating-room, and two dark rooms, all fitted up with every convenience. On this floor will be a large ward (30ft. by 20ft.) for men, with a bathroom and offices, a nurses' room, kitchen, pantry, and office. At the north end of the hall there will be a fine staircase, 5ft. wide, and a handsome window, which some day, it is hoped, will be filled with stained glass, the proposed subject being the Biblical one of "Christ Healing the Blind." The first floor provides a women's ward 30ft. by 20ft., night nurses' room, and four other bedrooms, which may be used as special wards if required. Bathrooms are provided, all of which are to be lined with cream-coloured glazed tiles. The architect is Mr. J. Buckley Wilson.

In the current number of the *Fortnightly Review*, Dr. Robson Roose examines the problem, What are the natural conditions, and what the personal precautions for securing longevity. Dr. Roose believes heredity to be the most powerful factor in causing longevity, and moderation in all things the best precaution. Marriage would appear to be conducive to longevity. A well-known French savant, Dr. Bertillon, states that a bachelor of twenty-five is not a better life than a married man of forty-five, and he attributes the difference in favour of married people to the fact that they take more care of themselves, and lead more regular lives. It must, however, be remembered that the mere fact of marrying indicates superior vitality and vigour, and the ranks of the unmarried are largely filled by the physically unfit. With respect to the learned professions, it would appear that among the clergy the average of life is beyond that of any similar class. Among lawyers there have been several eminent judges who attained a great age, and the rank and file of the profession are also characterised by a decided tendency to longevity. The medical profession supplies but few instances of extreme old age, and the average duration of life among its members is decidedly low—a fact which can be easily accounted for. Broken rest, hard work, anxieties, exposure to weather and to the risks of infection cannot fail to exert an injurious influence upon health.

The following vacancies are announced this week, the amount of the salary in each case being given after the name of the institution :

**Resident Medical Officers.**

Children's Hospital, Pendlebury. Junior Medical Officer. £80, board, &c.
Nottingham General Hospital. Senior Medical Officer.—£120, board, &c.
Chorlton-on-Medlock Dispensary. House Surgeon.—£100, rooms, &c.
Gravesend Hospital. House Surgeon.—£80, board, &c.
Derbyshire General Infirmary. House Surgeon.—Board, &c.
Birmingham General Hospital. Two Assistant House Surgeons.—Board, &c.
Clare Infirmary. Surgeon.—£94, rooms, &c.
Liverpool Dispensaries. Head Surgeon.—£200, board, &c.

**Non-Resident Medical Officers.**

University College, Liverpool. Demonstrator in Physiology. £120.
Dental Hospital, London, Medical School. Tutor.—£40.

A proposed hospital on Puffin Island sounds absurd, and one wonders where the patients are to come from. But the proposal is made in all seriousness, for some shelter is needed for sailors suffering from infectious diseases on ships which enter the Menai Straits. At present this quaint spot is used as a sort of menagerie by the Liverpool Biological Society, and perhaps they may object to having typhoid or smallpox introduced amongst their pet specimens.

Lately Lady Theodora Guest, on behalf of the Marchioness of Westminster, opened a new ward at the Weymouth and Dorset County Royal Eye Infirmary. Her ladyship read a communication which she had received from Lady Ampthill (lady-in-waiting to the Queen) expressing the deep interest her Majesty took in this institution, of which she is a patroness. The new wing has been erected by public subscription, the fund having been started during the Jubilee year.

The Committee of the Free Library, London-street, Bethnal Green, E., should direct their attention to an objectionable practice on the part of their officials. They write to an author asking him to present a free copy to the library of any new book he may publish. If he assents they then send him a bundle of prospectuses, and ask him to worry his friends as a sort of amateur canvasser on behalf of the library in question. Is it wise for the conductors of a free library like this to add insult to injury by first of all procuring a free copy of an expensive work, and then annoying the donor by urging him to tout for an institution which, though no doubt very useful, is still very limited in its aims and influence?

The members of the Vaccination Commission have been formally appointed, and the following questions given for their special consideration : (1) The effect of vaccination in reducing the prevalence of, and mortality from, smallpox. (2) What means, other than vaccination, can be used for diminishing the prevalence of smallpox ; and how far such means could be relied on in place of vaccination ? (3) The objections made to vaccination on the ground of injurious effects alleged to result therefrom ; and the nature and extent of any injurious effects which do, in fact, so result. (4) Whether any, and if so, what means should be adopted for preventing or lessening the ill effects, if any, resulting from vaccination ; and whether, and if so, by what means, vaccination with animal vaccine should be further facilitated as a part of public vaccination. (5) Whether any alterations should be made in the arrangements and proceedings for securing the performance of vaccination, and, in particular, in the provisions of the Vaccination Acts with respect to prosecutions for non-compliance with the law.

A correspondence has been going on in the *Nottingham Guardian* on the subject of permanent *v.* temporary hospitals for infectious diseases. The Corporation desires to spend £30,000 on a permanent hospital at Bagthorpe. A correspondent says that scarlet fever is the only infectious disease rife in Nottingham, and that mothers would not send their children all that way. "To-day," he writes, "the number of scarlet fever patients in the temporary hospital at Woodborough-road is 32. With the hospital at Bagthorpe, I think I am within the mark if I say three-fourths of these cases will be kept at home, leaving one-fourth, or eight cases—chiefly parish ones—to be sent to Bagthorpe. Let your readers fancy an hospital costing an initial expenditure of nearly £50,000, and several thousands a year to keep going, with only eight patients. And what of those times when there is no scarlet fever at all?" This is all another proof that the public do not like prevention, and yet cure is difficult when an epidemic seizes on a town.

The annual meeting of the Hospital for Epilepsy and Paralysis, Regent's Park, was held on Thursday at Willis's Rooms, the Rev. Canon Duckworth in the chair. Among those present were Lady Smith, Sir W. Magnay, Bart., Mr. W. L. Lawson, General Maclagan, Drs. Althaus and Ogilvie, Messrs. Drummond and Fisher, and others. After explaining the object of certain alterations in the rules, which were afterwards carried, Canon Duckworth referred to the foundation of the hospital twenty-two years ago, under a similar title to its present one, at a house in Charles-street, Portman-square, and stated that the increasing work thrown upon it had induced the committee, encouraged by two large anonymous donations, to take, some seventeen years since, the house facing the Regent's Park now occupied by the institution. Those who knew its site knew also that there was no site in London more eminently eligible, from its cheerful and airy situation, for a hospital undertaking the treatment of the diseases of the very peculiar type treated at this institution. His hearers would therefore share his regret at learning that in ten or eleven years the lease would expire, that the whole row of houses might (it was rumoured) be pulled down, and then where was the work of the hospital to be carried on? Far-seeing friends therefore thought that the time had come for raising a building fund for the erection of a suitable building on or near the present site if possible. In a recent visit to the hospital he had seen much that deeply interested him, and had an opportunity of seeing a new, and at first sight rather startling, mode of treating patients suffering from that terrible complaint—locomotor ataxy. It really struck one as a rehearsal for the gallows, for, as a matter of fact, it consisted in repeatedly suspending the patient for short periods by the head. The results were, however, most beneficial, and the treatment was sought for by out-patients coming from all parts, who found their worst symptoms alleviated. In moving the adoption of the annual report, General Maclagan, chairman of committee, urged strongly the need of largely increased (and of new) annual subscriptions. It was by recent legacies that the burden of debt had been removed, but no more uncertain and variable source of income than this could be named. He dwelt upon the excellent work done by the secretary, Mr. Howgrave Graham, not only in so largely increasing their receipts, but also by the great extension of sympathy and interest in the work of the hospital which his labours had evoked. The re-election of the retiring members of committee (Sir W. Magnay, Dr. Althaus, and Mr. Fisher) was moved by the hon. chaplain, the Rev. W. L. Lawson, in the unavoidable absence of Cardinal Manning, whose attendance had been expected. Dr. Althaus moved a vote of thanks to Canon Duckworth, which was cordially carried.

